# Overview of ICD-11 architecture and structure

**DOI:** 10.1186/s12911-021-01539-1

**Published:** 2022-05-16

**Authors:** Christopher G. Chute, Can Çelik

**Affiliations:** 1 Johns Hopkins Schools of Medicine, Public Health, and Nursing, 2024 E Monument St, Suite 1-200, Baltimore, MD 21287 USA; 2 World Health Organization, Geneva, Switzerland

**Keywords:** Medical classification architecture, Content model, Foundation, Linearization, International classification of diseases, ICD

## Abstract

**Background:**

The International Classification of Diseases (ICD) has progressed from a short list of causes of death to become the predominant classification of human diseases, syndromes, and conditions around the world. The World Health Organization has now explored how the ICD could be revised to leverage the advances in computer science, ontology, and knowledge representation that had accelerated in the twentieth and early twenty-first centuries.

**Methods:**

Many teams of clinical specialists and domain leaders worked to fundamentally revise the science and knowledge base of ICD-11. Development of the ICD-11 architecturally was a fundamental revision. The architecture for ICD-11 proposed in 2007 included three layers: a semantic network of biomedical concepts (Foundation), a traditional tabulation of hierarchical codes that would derive from that network (Linearization), and a formal ontology that would anchor the meaning of terms in the semantic network. Additionally, each entry in the semantic network would have an associated information model of required and optional content (Content Model).

**Results:**

This paper describes the innovative architecture developed for ICD-11.

**Conclusion:**

ICD11 is a revolutionary transformation of a century long medical classification that retains is historical rendering and interface while expanding the opportunity for multiple linearization and underpinning its content with a formally constructed semantic network. The new artifact can enable modern data science and analyses with content encoded with ICD11.

## Introduction

The International Classification of Diseases (ICD) has progressed from its mid-19th-century origins as a short list of causes of death to become the dominant classification of human diseases, syndromes, and conditions around the world [1]. Despite a profound increase in content and impact over more than a century between the initial versions of the International Statistical List and the release of the 10th revision of the ICD (ICD-10) in 1990, there was virtually no evolution of the structure or architecture of the classification. It remained little more than a table of terms with associated code values. Some have characterized the ICD as a 16th-century spreadsheet [2], harkening to the structure of the ICD’s ancient predecessor, the London Bills of Mortality [3], established during the reign of King Henry VIII of England.

The leadership of the Classifications and Terminology team at the World Health Organization (WHO) were very much aware of the shortcoming of the ICD in the modern digital age. Around 2005 they organized meetings to explore how the ICD could be revised to leverage the advances in computer science, ontology, and knowledge representation that had accelerated in the twentieth and early twenty-first centuries. From its inception, the next revision of the ICD—ICD-11—was intended to leapfrog classification tradition and embrace the modern digital revolution. The only question was how to reconcile that brave new vision with the traditional needs and requirements of the statistical mortality and public health communities, who had deep dependencies on the centuries of evolved structure.

## Methods

ICD-11 was developed by many teams of clinical specialists and domain leaders in a series of 19 topic advisory groups (TAGs). Each TAG and their corresponding sub-groups comprised froma dozen to more than a score of experts, collaborating to fundamentally revise the science and knowledge base of ICD-11. Relevant to this paper was the creation of a special Informatics TAG, which contributed greatly to the ICD-11 architectural development. This TAG raised informatics and the computational design of ICD-11 as a peer priority alongside clinical domains.

The architecture for ICD-11 proposed in 2007 included three layers: a semantic network of biomedical concepts (Foundation), a traditional tabulation of hierarchical codes that would derive from that network (Linearization), and a formal ontology that would anchor the meaning of terms in the semantic network. Additionally, each entry in the semantic network would have an associated information model of required and optional content (Content Model).

### Results

#### The content model

The core content model of the ICD expands greatly upon the simple historical term and code structure in previous ICD versions. Table 1 shows the possible elements for each term or concept in the ICD. Some elements are required and fully populated in the released version. The remaining elements are variously complete, and remain an opportunity for future work. Nevertheless, ICD-11 terms have substantial clarity, with fully specified terms and definitions. They also exhibit permanence with unchanging uniform resource identifiers, or URIs.

**Table 1:** Elements of the information model for each term in the Foundation

Element	Description
*Concept title	The preferred name for a concept. This can vary by language
*Unique identifier (URI)	A permanent identifier assigned by the WHO that will never change
*Fully specified name	A complete name that describes the concept. It explicitly does not make assumptions about a child concept inheriting the context and meaning of a parent
*Synonyms	Alternative names for the concepts. These vary by language
Classification properties	Whether a concept is a disease, syndrome, symptom, finding, or health condition
*Parent and child relationships	Linkages to parent terms (in the Foundation, there can be more than one parent) and all of its immediate child terms. This allows for the creation of an acyclic-graph semantic network
*Brief definition	A short definition of the term
Long description	A more complete definition that may include related observations
Body system	The anatomical locations where the condition does or can occur
Manifestations	Signs and symptoms of the condition
Etiology	Causes of the disease (e.g., bacterial organisms or genomic causes)
Genomic association	Genomic characteristics that modify the risk for disease
Severity	Specific severity levels, stages, or grades, and their association with an extension code
Temporality	Acute vs. chronic, as well as life cycle of the condition if appropriate
Functional impact	Functional consequences of a disease or condition, such as blindness

^*^Required element

### The foundation

**Fig. 1:**
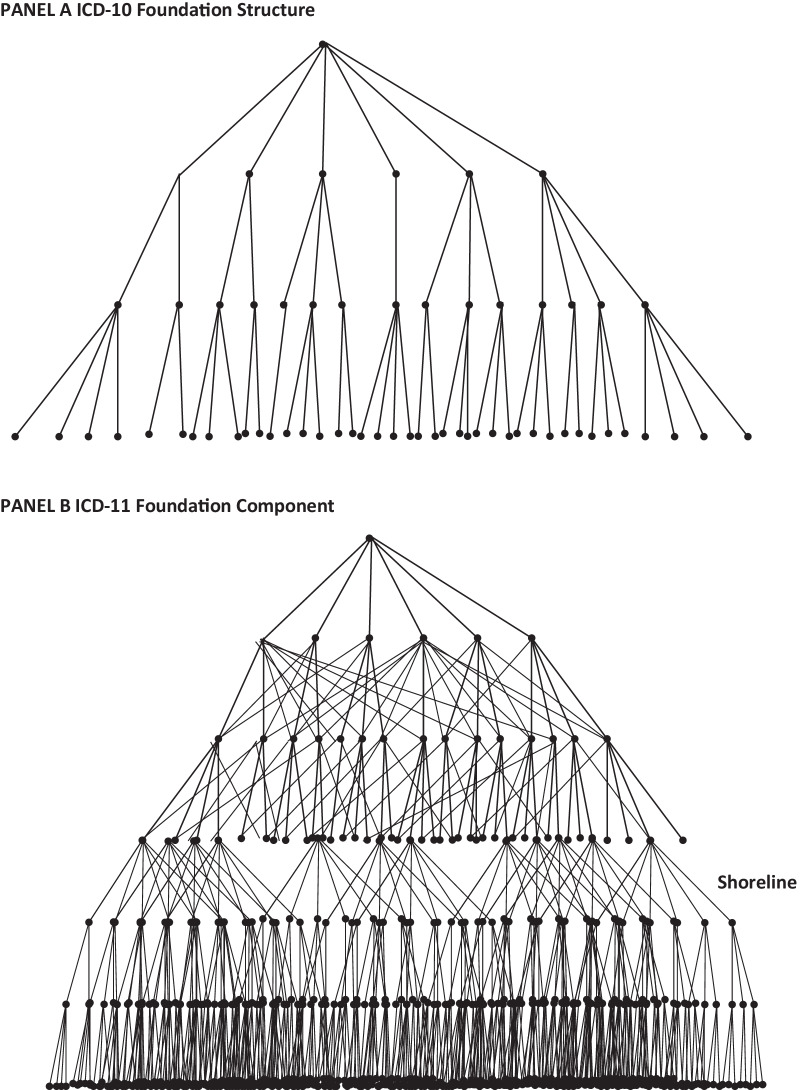
**a** Schematizes the relatively shallow, strict hierarchy of ICD-10. **b** Illustrates the multiple inheritance (a concept may have more than one parent, and thus is not mutually exclusive), as well as the greater relative depth of ICD-11

All concepts in the ICD are rendered in the Foundation, which is an acyclic graph (meaning no concept can ever be its own descendant as or parent) of all concepts and their relationship trees. Unlike in the historical ICDs, the Foundation may have multiple inheritances, where a single term may have one or more, sometimes many more, conceptual parents. Thus, stomach cancer is a child in the cancer tree as it has been for decades, but it is also a child in the gastrointestinal illness chapter, where it was previously absent. This allows for the assertion of ontological structures and supports complex navigation in the Foundation, which was impractical in earlier versions of the ICD (Fig. 1). The depth of this semantic network is virtually unlimited, meaning highly specialized conceptual children can exist in this network without any limitation of digits in a coding structure. It can be vastly larger than any historical version of the ICD, since this is not the system used for practical disease coding. It is the conceptual underpinning of the entire ICD-11 system.

Relationships between parents and children are mostly expressed as a subset of predicate logic formalisms, such as those in Web Ontology Language (OWL) [4]. However, there are some exceptions to this in the Extension Codes chapter. Because of this the developers opted to invoke a more simple “broader-than” or “narrower-than” set of relationships, as described in the Simple Knowledge Organization System framework [5] when the classifications are made available in the ICD’s application programming interface (API) [6]. This supports an unambiguous assertion of relations between conditions to create hierarchy but does not impose the absolute logic requirements of OWL in every case.

### Linearizations

**Fig. 2:**
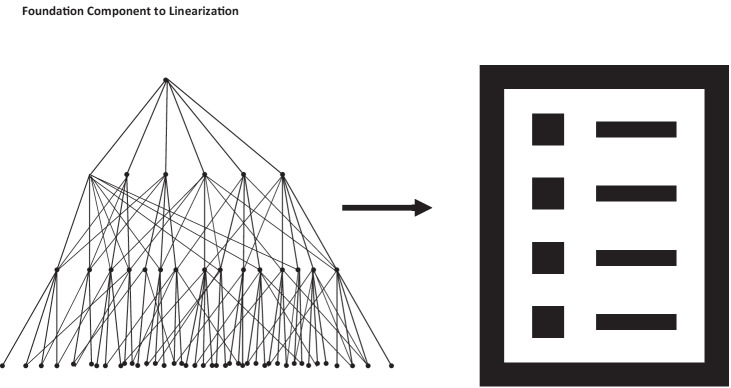
A schematic depiction of how the multiple inheritance semantic network of the Foundation is “linearized” into a mutually exclusive, strict hierarchy than can be rendered as a non-overlapping list of rubric codes and descriptions

The Foundation does violate a key precept of statistical classifications, which is that the content must be mutually exclusive and exhaustive. Mutually exclusive concepts in practice mean that they must have only one place in a concept hierarchy, and must therefore have only one parent term. Exhaustive classifications are achieved by additional residual categories such as “other” or “not specified” at the terminus of concept branches. Because this is not the architecture of the Foundation, linearizations that would have these properties were derived from the Foundation. These linearizations are effectively a strict “walking in a line” of the Foundation concept tree to a limited depth by deliberately choosing a single parent for inheritance; they thus satisfy the requirements of being mutually exclusive and exhaustive (Fig. 2).

**Fig. 3:**
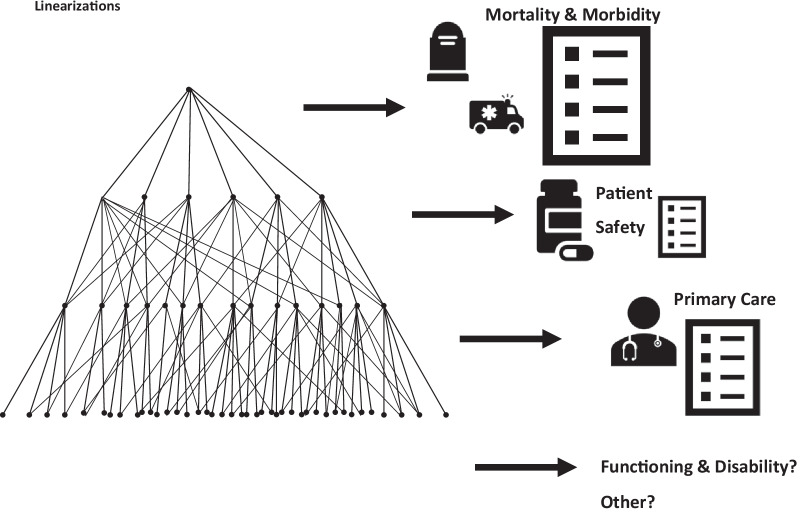
The process of linearizations can be accomplished repeatedly, choosing different parents from the Foundation as the primary or linear parent, to achieve mutually exclusive statistical classifications or linearizations for a spectrum of use-cases

In an interesting extension, ICD-11 can support multiple, simultaneous linearizations from the Foundation. These include the main tabular publication of the Morbidity and Mortality Statistics linearization, as well as general linearizations for primary care and, for example, a subspecialty linearization in dermatology (Fig. 3).

#### Residual categories

To ensure that linearizations are exhaustive, the addition of Other (equivalent to “Not Otherwise Specified”) or Other Specified (equivalent to “Not Elsewhere Classified”) are algorithmically added to linearization branches at all levels. Linearization authors may specify in the Foundation that some categories should not have these residuals algorithmically added where they do not make sense. Since these residual categories have meaning and context only within the linearization in which they appear, they will not otherwise have any reference in the Foundation.

#### The shoreline

The Foundation can and does descend to an arbitrary depth. However, a linearization is rendered in a strict hierarchy, where the terminal leaves are typically residual terms. These trees are numbered with a hierarchical coding system of fixed digits; the Morbidity and Mortality for Statistics is the main linearization, where the hierarchical codes have seven alphanumeric digits. Developers of linearizations in the classification have two major options for representing concepts: making more specific or meaning-qualified children, or leaving such specificity and qualification of meaning to post-coordinated expressions. The Foundation, by design, attempts to capture the specific terms and qualified meanings that are referenced in clinical medicine, to an arbitrary level of detail. Not all of those terms can “fit” in a fixed-digit hierarchy, and are thus not explicitly rendered in a linearization; they are accommodated with post-coordination. The Foundations functions as a deep sea of terms and meanings, where only a subset of the most common or important terms can appear on the metaphorical landmass of the linearization. The more specific terms, while expressible via post-coordination, are said to be “below the shoreline” of that linearization, in the depths of the Foundation.

#### Post-coordination

The content of the Foundation provides an enormously rich thesaurus for the ICD, and in fact the functional index of ICD-11 is built from the Foundation. However, even greater expressivity can be achieved by the combination of base terms, such as a disease entity, with qualifier codes. ICD-11 contains a complete chapter of such qualifier or extension codes, which can be combined with terms to compose “sentences” of clinical description. Thus, a given cancer can be modified to include histology, anatomic site, stage, and extent as a single ICD-11 assertion. This compositional structure enables profoundly granular, detailed, and specific descriptions of clinical entities. ICD-11 effectively marries the aggregation capacity of a clinical classification with the potential of a highly expressive terminology. (Post-coordination is explored more fully in a companion paper in this special issue [7]).

#### Sanctioning rules

ICD-11 contains detailed information on which entities can be extended by using post-coordination axes in order to prevent meaningless combinations. In many cases the value sets of these axes are also limited to an area that makes sense to the disease. For example, the gastric cancer code can be enhanced by using anatomical detail, but this detail is limited to related subtrees of the anatomy hierarchy.

The capacity to create post-coordinated expressions may result in a user creating an expression that already exists as a pre-coordinated expression in a linearization. Thus, each linearization includes tables of pre-coordinated expressions and their post-coordinated equivalents, called sanctioning rules. If a user attempts to post-coordinate an expression where the equivalent pre-coordinated expression exists, coding software would invoke these sanctioning rules to generate the correct final code. Otherwise, the classification would risk violating the mutually exclusive principle of statistical classification, which allows one and only one way of capturing a clinical condition to avoid double counting or misclassification. (This is described more fully in the paper on post-coordination [7]).

### The ontology layer

To computationally anchor the unambiguous meaning of terms in the Foundation, the developers envisioned creating or adapting an ontology layer built with a formal description logic, such as OWL, and linking those terms to the Foundation more informally through simple knowledge organization system, or SKOS, principles. Substantial preliminary work was done on this effort, convincingly demonstrating the principle [4–8].

Currently, more than 3000 entities in ICD-11 are formally defined, and these definitions are used to figure out post-coordination/pre-coordination equivalences. However, the work on the ontology layer was not completed and remains an opportunity for future development.

### ICD-11 tooling and the ICD-API

ICD-11 comes with a set of software tools [9]. ICD-11 Browser is multilingual web-based software that allows users to browse the classification. ICD-11 Coding Tool is another multilingual tool that is specifically customized for medical coding with ICD-11. It has features such as word completion and word suggestion, and it is powered by a flexible search engine that is capable of working with post-coordination combinations.

In addition to the software provided to support the use of the classification, ICD-11 comes with a powerful API [10]. Software developers could use this API to integrate ICD-11 and related functionality into their software in a facile fashion. ICD-API not only provides access to the full detail of the classification, but also allows users to benefit from other related functionality, such as the search engine that is behind the ICD-11 Coding Tool. ICD-API can be leveraged through any software written in any programming language. It can be accessed online or deployed locally. Embedded Coding Tool, another software component made available to software developers, makes it easy to add ICD-11 Coding Tool functionality to any web-based software.

### Impact

We believe these architectural changes enable advantages such as the following:

- Enhanced navigation through structure and software
- A digital framework for enabling concept browsing, linkage, and context
- Support for semi-automated coding
- Vastly larger base of concepts in Foundation (~ 70 k)
- Facile integration with health applications and electronic records via API
- Function as a first-rank data science resource for research

## Conclusion

The architecture of ICD-11 represents a twenty-first century rendering of knowledge, data, and concepts in a manner that enables substantial computer support for applying the classification. The suite of tooling provided for browsing and coding depends upon these architectural features. Further, the elegant structures can further mature to embrace deeper computer coding assistance, including automated coding suggestion directly from electronic patient records. A goal of ICD-11 development was to embrace modern computing principles and structures that can support continued development and sophistication into the future.

## Data Availability

Not applicable.

## Abbreviations

API: Application programming interface
ICD: International classification of diseases
ICD-10: International classification of diseases, 10th revision
ICD-11: International classification of diseases, 11th revision
OWL: Web ontology language
SKOS: Simple knowledge organization system
TAG: Topic advisory group
URI: Uniform resource identifier
WHO: World Health Organization
